# Women empowerment in reproductive health: a systematic review of measurement properties

**DOI:** 10.1186/s12905-021-01566-0

**Published:** 2021-12-20

**Authors:** Maryam Vizheh, Salut Muhidin, Zahra Behboodi Moghadam, Armin Zareiyan

**Affiliations:** 1 Department of Reproductive Health and Midwifery, School of Nursing and Midwifery, Tehran University of Medical Sciences, Tehran, Iran; 2 Department of Management, Macquarie Business School, Macquarie University, Sydney, NSW 2109 Australia; 3 Public Health Department, Health in Disaster & Emergencies Department, Nursing Faculty, Aja University of Medical Sciences, Tehran, Iran

**Keywords:** Women empowerment, Reproductive health, Measurement, Psychometrics, Validity, Reliability

## Abstract

**Introduction:**

There is a considerable dearth of official metrics for women empowerment, which is pivotal to observe universal progress towards Sustainable Development Goals 5, targeting "achieve gender equality and empower all women and girls.” This study aimed to introduce, critically appraise, and summarize the measurement properties of women empowerment scales in sexual and reproductive health.

**Methods:**

A comprehensive systematic literature search through several international electronic databases, including PubMed, Scopus, Embase, ProQuest, and Science Direct was performed on September 2020, without a time limit. All studies aimed to develop and validate a measurement of women empowerment in sexual and reproductive health were included. The quality assessment was performed through a rating scale addressing the six criteria, including: a priori explicit theoretical framework, evaluating content validity, internal consistency, and factor analysis to assess structural validity.

**Results:**

Of 5234 identified studies, fifteen were included. The majority of the studies were conducted in the United States. All studies but one used a standardized measure. Total items of each scale ranged from 8 to 23. The most common domains investigated were decision-making, freedom of coercion, and communication with the partner. Four studies did not use any conceptual framework. The individual agency followed by immediate relational agency were the main focus of included studies. Of the included studies, seven applied either literature review, expert panels, or empirical methods to develop the item pool. Cronbach's alpha coefficient reported in nine studies ranged from α = 0.56 to 0.87. Most of the studies but three lack reporting test–retest reliability ranging r = 0.69–0.87. Nine studies proved content validity. Six criteria were applied to scoring the scales, by which nine of fifteen articles were rated as medium quality, two rated as poor quality, and four rated as high quality.

**Conclusion:**

Most scales assessed various types of validity and Internal consistency for the reliability. Applying a theoretical framework, more rigorous validation of scales, and assessing the various dimensions of women empowerment in diverse contexts and different levels, namely structural agency, are needed to develop effective and representing scales.

## Introduction

Recognition and measurement of women empowerment are critical for global development and human rights [1]. This was accentuated as the Sustainable Development Goal (SDG 5), which targets to "achieve gender equality and empower all women and girls” [1].

Although the growing body of literature addresses the impact of women empowerment on reproductive outcomes, it is only recently that reproductive empowerment was explicitly distinguished as a distinct dimension of empowerment itself [2, 3]. Edmeades et al. (2018) proposed the following definition of reproductive empowerment according to a recently developed framework: “Both a transformative process and an outcome, whereby individuals expand their capacity to make informed decisions about their reproductive lives, amplify their ability to participate meaningfully in public and private discussions related to sexuality, reproductive health, and fertility, and act on their preferences to achieve desired reproductive outcomes, free from violence, retribution or fear” [2].

Reproductive and sexual empowerment is critical because, in many contexts, intimate relationships frequently occur between individuals with vastly unequal power. In many cultures, normative expectations toward gendered heterosexual sex roles and gender inequalities negatively influence women’s sexual power and restrict their ability to negotiate sexual matters with male partners [4].

The literature review showed that scales of women empowerment in reproductive health, especially in the past years, concentrated more on “power”, where power structures limit women’s sexual and reproductive health capabilities. These measures such as Sexual Assertiveness Scale [5], The Sexual Relationship Power Scale [6], and the Sexual Pressure Scale [7, 8], mainly addressed the experience of pressure and coercion regarding sexual activity, sexual desires, HIV/AIDS (Human immunodeficiency virus/Acquired immunodeficiency syndrome) risk and prevention, and STD (sexually transmitted diseases) prevention to better capture the gender norms and dynamics shaping women’s sexual decisions and outcomes. The recent measures, on the other hand, recognize “choice” as a critical component of empowerment. Kabeer's foundational work on women's empowerment in the early 2000s, and then developing a framework by World Bank [9, 10], introduced decision-making and exercise of choice as the components of the agency. These concepts were used commonly to design scales of women empowerment measurements such as Sexual and reproductive empowerment [11], Reproductive Decision-making Agency [12], Women’s and Girls’ Empowerment in Sexual and Reproductive health (WGE-SRH) [13], aimed to assess women’s agency in decision making over vital sexual and reproductive health matters.

The lack of standardized terminology and measurements of women reproductive empowerment in addition to the conceptual ambiguity have directly influenced implications for its measure. Consequently, there is considerable variability in the association between reproductive empowerment and health outcomes. This restrains policymakers and authorities from planning effective interventions to improve reproductive empowerment or reproductive outcomes [3].

Although it is suggested that gender-based control in hetro-sexual relationships is correlated with sexual and reproductive consequences [14], there is a considerable dearth of official metrics for women empowerment, which is pivotal to observe progress towards SDG 5 [1]. Demand for developing standard measurements of women empowerment would be more highlighted given that adequate data for 80% of indicators to monitor SDG5 is lacking, often due to the absence of valid measures [15]. This study aimed to introduce, critically appraise, and summarize the quality of the women empowerment’s measurement properties in sexual and reproductive health.

### Conceptual framework

In this review, we applied the Kabeer framework where conceives empowerment as the ability of exercise choice consisting of three inter-related dimensions including (1) resources, defined as not only access to but the future claims to material, human and social resources; (2) agency, including decision making, negotiation, deception, manipulation, subversion and resistance; and (3) achievements encompasses well-being outcomes which are sexual and reproductive health [9]. We also used the conceptual framework of women reproductive empowerment proposed by Edmeades, Mejia, and Sebany (2018). Within this approach, reproductive empowerment results from the interaction of three interrelated, multi-level processes: voice, choice, and power. Voice indicates women’s capacity to exercise their reproductive goals, interests, and desires and have meaningful participation in reproductive decision making. Choice implies the ability of women to make a meaningful contribution to reproductive decisions.Power indicates the ability to shape the process of reproductive decision-making by exerting power over others [3]. Power operates at multiple levels, including couple level, families level, and expanding to the community and societal levels [13].

Combining both frameworks, we sought psychometric assessment studies that had chosen any of the abovementioned concepts and considered sexual and reproductive health as the main outcome.

## Methods

### Study design and search strategy

Preferred Reporting Items for Systematic Reviews and Meta-Analyses (PRISMA) guidelines [16] were used to conduct the current review. A comprehensive literature search was carried out to identify women empowerment scales used in sexual and reproductive health and their properties. The first author (M.V) performed the systematic review on September 2020, in several international electronic databases, including PubMed, Scopus, Embase, ProQuest, and Science Direct, without a time limit. Various search strategies involving keywords, index/subject terms, and Medical Subject Headings (MeSH) terms were used. A brief sample of keywords included: (women, female, girls) AND (reproduction, sexual, family planning, family planning services, fertility, contraception, birth spacing, birth intervals) AND (empowerment, power, agency, decision-making, autonomy, coercion, choice, negotiation, mobility) AND (measurement, scale, instrument, tool, questionnaire, indicator) AND (psychometric, validity, validation study, reliability, reproducibility of results). Moreover, Google Scholar and the references of the included articles were reviewed manually.

### Inclusion and exclusion criteria

All studies aimed to develop a new scale or adapt an existing scale and reported the results of the reliability and validity testing were included in this research. Studies that developed and validated a women empowerment scale but the primary outcome were not the sexual and reproductive health were excluded. Moreover, studies that used a new scale without performing a psychometric analysis were excluded, too. Studies published in a language other than English and non-peer-reviewed reports, books, and dissertations, were excluded.

### Outcome of interest

In the aim of this review, the construct of interest is “women reproductive empowerment” the population of interest is “women and girls”, the type of scale of interest is “all” either self-report questionnaire or interviewer-administered, and “all” measurement properties were evaluated in the review.

### Data extraction

The first author (M.V) screened all titles and abstracts from the search results. After identifying all relevant articles, full texts were reviewed by all authors. Two authors (M.V and A.Z) contributed to extracting the data from the included studies. Characteristics of the study samples and scales and the measurement properties were extracted. The qualitative data analysis was chosen to synthesize data in this study for two reasons. First, we found a large degree of heterogeneity between studies by examining the study characteristics, including population features, methods of determining construct validity, different domains addressed in scales, etc. Second, considering the purpose of this study to introduce, critically appraise, and summarize the measurement properties of relevant scales, the authors decided not to use quantitative data analysis, which has little implication.

### Assessment of methodological quality

Assessing the methodological quality of included studies was performed by two authors (M.V and A.Z) separately. Both authors discussed the ranking system to ensure its accuracy. The differences between them, either in data extraction or quality rating, were solved by another author, Z.BM.

Methodological quality was evaluated through three dimensions, including the developments of items, validity, and reliability. To evaluate item development, we assessed whether a literature review, empirical study, or expert panel were conducted to develop the measurement. Assessing reliability focused on whether internal consistency and test–retest reliability were determined. Validity was assessed by examining the methods used to determine content validity (the degree to which the content scale reflects the construct); structural validity (the degree to which the scores on the scale represent the dimensionality of the construct); internal construct validity (the consistency between scales and hypothesis); and external construct validity (whether measures of constructs strongly correlate or minimally correlate with one another in the hypothesized way” (Table 2) [17, 18].

A rating scale applied in some systematic reviews was used to evaluate the quality of the scales’ measurement properties [17, 19]. Six criteria were the basis of the scoring, including whether studies used a priori explicit theoretical framework; assessed the content validity; assessed the internal reliability scores (α > 0.7), determined the structural validity using exploratory factor analysis; determined the internal construct validity through confirmatory factor analyses; and assessed the external construct validity or not. The scores on each item range from 0 (none of all six criteria were fulfilled) to 6 (all of six criteria were fulfilled). The total score of study ≤ 2 interprets as poor quality; 3–4 means medium quality, and the total score ≥ 5 is considered high quality (Table 3).

## Results

### Study characteristics

**Fig. 1:**
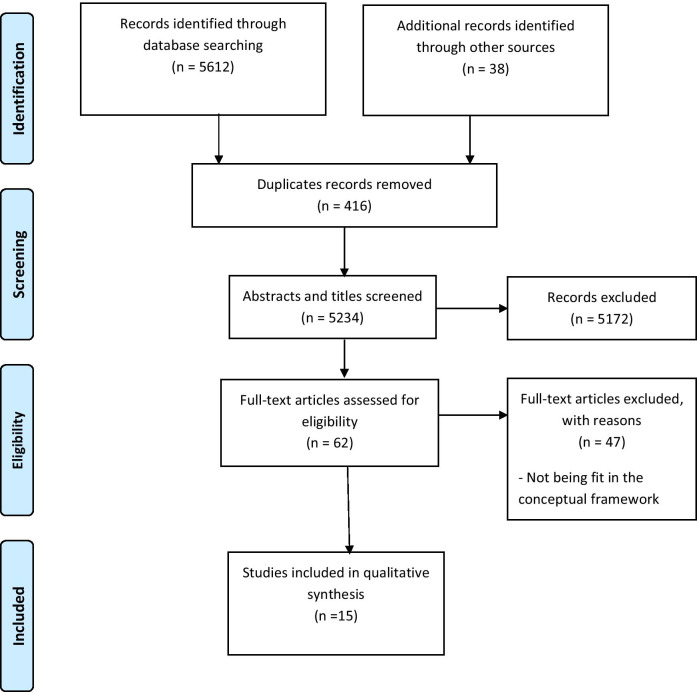
PRISMA flow diagram of study process

The search strategy yielded 5234 relevant records. Finally, 62 full texts were reviewed, of which 15 separate scales were identified (Fig. 1).

Ambiguous scales that measured the components, dimensions, or subscales of women empowerment but did not fit in our framework and original search strategy were excluded to consistently adhere to our conceptual framework (n = 46). Another full text aimed at the psychometric analysis of Reproductive Agency Scale 17 (RAS-17), composing pregnancy-specific and non-pregnancy-specific agency items among Qatari and non-Qatari women with a normal pregnancy [20], was excluded to achieve the maximum homogeneity of the results. Some scales such as the Survey-Based Women’s Empowerment (SWPER) Index and Composite Women’s Empowerment Index (CWEI) have been developed to measure women empowerment [21, 22]; however, they did not include in this review because they were not applicable in sexual or reproductive health.

A detailed description of the included scales is shown in Table 1. The results revealed that included articles did not represent diverse geographical areas. The majority of studies (8/15) were conducted in the United States [5–8, 23–26]. Two were done in Nepal [12, 27], one in Spain [28], and the rest of the studies (4/15) were carried out in African countries [13, 29–31]. The sample size varied from 235 to 4674 in primary studies and 111,368 in one study using the Demographic and Health Surveys (DHS). The age of participants ranged between 16 and 71. The items of each scale ranged from 8 to 23. The target population in studies were as following: three studies (3/15) included adolescents and young adults (15–24 years) [11, 29, 30], three (3/15) were carried out on young women aged 16–29 [7, 8, 25]; one conducted in young women 20–35 years [12]; six studies (6/15) aimed to assess women in reproductive age defined as those aged 15 to 49 years [5, 6, 13, 26, 27, 31]. Two studies extended the age group of participants beyond 45 years; in one study, women at the ages of 15 to 60 [24]; and in another, women ages 18 to 71 were included [32].

**Table 1:** Characteristics of included studies

Author, year Country	Construct	Items/subscales	Target population in quantitative surveys	Conceptual framework	Measured outcomes	Dimensions of women empowerment	Internal consistency (Cronbach's alpha)
Upadhyay et al. 2020 [11] USA	Sexual and reproductive empowerment	23 items/7 subscales: – Comfort talking with a partner – Choice of partners, marriage, and children – Parental support – Sexual safety – Self-love – Sense of future – Sexual pleasure	1117 Adolescents and young adults aged 15–24	Kabeer’s framework	– Using the desired contraceptive method – Access to sexual and reproductive health services – Access to health information	Individual agency/immediate relational agency	0.80
Upadhyay et al. 2014 [24] USA	Reproductive Autonomy Scale	14 items/3 subscales – Freedom from coercion – Communication – Decision-making	1892 women aged 15–60	Theory of gender and power developed by Connell	– Current use of modern contraception – Reaching one’s reproductive desires and intentions – Unmet need for contraception	Individual agency	0.78 subscales: 0.65–0.82
Hinson et al. 2019 [12] Nepal	Reproductive Decision-making Agency	12 items – Agency around when to have children – Agency around whether to use contraception – Agency around which method of contraception	935 women aged 15–49	Reproductive empowerment framework, developed by Edmeades et al.	– The time of having children – Using family planning methods – Choosing the method of family planning	Individual agency	0.6416
Moreau et al. 2020 [13] Ethiopia, Uganda, and Nigeria	Women’s and Girls’ Empowerment in Sexual and Reproductive health (WGE-SRH)	14 items/3 subscales – Sexual existence of choice – Contraceptive existence of choice – Pregnancy existence of choice	1229 women aged 15–49	– The World Bank’s Empowerment Framework – The SRH Empowerment	– Volitional sex – Contraceptive use – Pregnancy by choice	Individual agency	0.56–0.79 For various subscales
McCauley et al. 2017 [25] USA	The Reproductive Coercion Scale (RCS)	9 items/2 subscales: – Pregnancy coercion – Condom manipulation	4674 women aged 16–29	None	Unwanted pregnancy	Immediate relational agency	–
Morokoff et al. 1997 [5] USA	Sexual Assertiveness Scale (SAS)	18 items/3 subscales – Assertiveness regarding initiation of sex – Assertiveness regarding the refusal of sex – Pregnancy/STD prevention	The first sample: 260 and 136 The second sample: 240 and 263, women at reproductive age	General conceptualization of assertiveness based on human rights to autonomy	– Unwanted sex – Pregnancy/STD prevention	Individual agency	0.82
Santos Iglesias and Carlos Sierra 2010 [32] Spain	Hurlbert Index of Sexual Assertiveness	19-item – Initiation of sex – No shyness/refusal of sex	400 men and 453 women (N = 853) 18 to 71 years	None	Sexual desires	Individual agency	0.87
Loshek and Terrell 2014 [26] USA	The Sexual Assertiveness Questionnaire (SAQ)	18 items – Satisfaction – Refusal – Risk-history	725 women aged 18–49	None	– Communication – Unwanted sexual acts	Individual agency	0.78 to 0.81 For various subscales
Jones 2006 [7] USA	The Sexual Pressure Scale (SPS)	19 items/5 subscales – Condom fear – Sexual coercion – Women’s sex role – Men expect sex – Show trust	306 urban women, aged 18 to 29	Gender stereotypical expectations	Sexual choices	Immediate relational agency	0.81
Jones and Gulick 2009 [8] USA	Sexual Pressure Scale for Women-Revised (SPSW-R)	18-item/subscales – Show trust – Women’s sex role – Men expect sex – Sex coercion	325 urban women aged 18–29	Gender stereotypical expectations	Sexual choices	Immediate relational agency	0.86
Pulerwitz et al. 2000 [6] USA	The Sexual Relationship Power Scale (SRPS)	23-item – Relationship control – Decision-making dominance	Women (N = 380 Women 18–45 years old	– The theory of gender – Power and Social Exchange Theory	HIV/AIDS risk and prevention	Immediate relational agency	0.84 for English version, 0.88 for Spanish version
Pulerwitz et al. 2018 [29] Kenya	The Sexual Relationship Power Scale (SRPS)	15-item	1101 adolescent girls and young women (AGYW) aged 15–24	The theory of gender – Power and Social Exchange Theory	HIV/AIDS risk and prevention	Immediate relational agency	15-item SRPS: 0.81 and 12-item SRPS-M: 0.76
Bhandari et al. 2014 [27] Nepal	Women’s Autonomy Measurement Scale	23-item – Decision making autonomy – Financial autonomy – Freedom of movement	250 Women at reproductive age	None	Maternal Health care Service Utilization	Individual agency	0.84
Kalysha Closson 2019 [30] South Africa	Sexual Relationship Power equity	Adaptation of Pulerwitz’s SRPS 8-items for women	235 young men and women aged 16–24	Theory of gender and power developed by Connell	HIV-risk factors	Immediate relational agency	0.63
Asaolu et al. 2018 [31] 19 countries representing/4 African regions	Women’s empowerment	4 items allocate to the subscale of health dimension – Access to healthcare domain	111,368 women aged 15–49	Kabeer’s framework	Access to healthcare	Structural agency	–

The most common domains of women empowerment in reproductive health that had been measured were: freedom from coercion, decision-making, communication with the partner, choice, control, autonomy, and ability to negotiate. “Kabeer’s framework of empowerment” was applied as the empowerment framework in two studies (2/15) [11, 31]; “The theory of gender and power” developed by Connell in four studies (4/15) [6, 24, 29, 30]; and “Sex scripts” (gender-stereotypical expectations to engage in sexual behavior) was used in two studies (2/15) [7, 8]. Moreover, the “Reproductive empowerment framework” developed by Edmeades et al. (2018) and “General conceptualization of assertiveness based on human rights to autonomy”, each one was used in one study [12]. The “World Bank’s Empowerment Framework” and “The sexual and health empowerment framework” developed by the authors were used in a study conducted by Moreau et al. [13]; whereas the rest of the studies did not apply any specific empowerment framework.

### Reliability and validity testing

Of the included studies, seven applied either literature review, or expert panels, or empirical method to develop the item pool (Table 2). Adequate internal consistency defined as the alpha > 0.7 was reported in nine studies (9/15). However, in four studies, poor internal consistency (α < 0.70) was seen. Two studies also did not report internal consistency. Most of the studies but three lack reporting test–retest reliability. Nine studies proved content validity. Six criteria were applied to score scales by which nine of fifteen articles were rated as medium quality, two rated as poor quality, and four rated as high quality (Table 3).

**Table 2:** Quality assessment of included studies (Methods adopted in the development of the scales included in the review (marked as ✓ or x))

Author, year	Item development				Reliability		Validity				
	Panel of experts	Qualitative interview with target	Literature review	Empirical study	Internal consistency	Test–retest reliability	Content validity	Face validity	Structural	Internal construct validity	External construct validity
Upadhyay et al. 2020 [11]	✓	✓	✓	✓	✓	–	✓	✓	✓	✓	–
Upadhyay et al. 2014 [24]	✓	–	✓	✓	✓	–	✓	✓	✓	✓	–
Hinson et al. 2019 [12]	✓	✓	✓	✓	✓		✓	✓	–	–	✓
Moreau et al. 2020 [13]	✓	✓	–	✓	✓	–	✓	✓	–	✓	–
McCauley et al. 2017 [25]	✓	✓	–	–	–	–	–	–	–	✓	–
Morokoff et al. 2010 [5]	–	✓	–	✓	✓	✓	–	–	✓	–	✓
Santos-Iglesias and Carlos Sierra 2010 [32]	–	–	–	–	✓	–	✓	✓	✓	✓	✓
Loshek and Terrell 2014 [26]	–	–	–	✓	✓	–	✓	✓	✓	✓	✓
Rachel Jones 2006 [7]	–	–	✓	✓	✓	✓	✓	✓	✓	✓	✓
Jones and Gulick 2009 [8]		✓	–	✓	✓	–	–	–	✓	✓	✓
Pulerwitz et al. 2000 [6]	–	✓	✓	✓	✓	–	–	✓	–	–	–
Pulerwitz et al. 2018 [29]	–	–	–	✓	✓	–	–	–	✓	✓	–
Bhandari et al. 2014 [27]	✓	–	✓	✓	✓	✓	✓	✓	✓	✓	✓
Closson et al. 2019 [30]	✓	–	–	✓	✓	–	✓	✓	✓	✓	–
Asaolu et al. 2018 [31]	–	–	✓	✓	–	–	–		✓	✓	✓

**Table 3:** Quality assessment of included studies (Ratings for each of the scales included in the review (1 if done and 0 if not done))

Author, year	Followed an a priori explicit theoretical framework	Reported efforts towards content validation	Exploratory factor analysis	Confirmatory factor analysis	Relationships with theoretically related construct (external construct validity)	Reliability scores above 0.7	Total score	Interpretation, ≤ 2 = poor quality; 3–4 = medium quality; 5–6 = high quality
Upadhyay et al. 2020 [11]	1	1	1	0	0	1	4	Medium quality
Upadhyay et al. 2014 [24]	1	1	1	0	0	1	4	Medium quality
Hinson et al. 2019 [12]	1	1	1	0	1	0	4	Medium quality
Moreau et al. 2020 [13]	1	1	1	0	0	1	4	Medium quality
McCauley et al. 2017 [25]	0	0	1	0	1	1	2	Poor quality
Morokoff et al. 2010 [5]	1	0	0	1	1	1	3	Medium quality
Santos-Iglesias and Carlos Sierra 2010 [32]	1	1	1	1	1	1	6	High quality
Loshek and Terrell 2014 [26]	1	1	1	1	1	1	6	High quality
Jones 2006 [7]	1	1	1	0	1	1	5	High quality
Jones and Gulick 2009 [8]	1	0	1	1	1	1	5	High quality
Pulerwitz et al. 2000 [6]	1	0	0	0	0	1	2	Poor quality
Pulerwitz et al. 2018 [29]	0	0	1	1	0	1	3	Medium quality
Bhandari et al. 2014 [27]	0	1	1	0	1	1	4	Medium quality
Closson et al. 2019 [30]	1	1	1	1	0	0	4	Medium quality
Asaolu et al. 2018 [31]	1	0	1	1	1	0	4	Medium quality

### Summary of included measures

#### Sexual and Reproductive Empowerment Scale

Sexual and Reproductive Empowerment Scale is a 23-item questionnaire developed and validated by Upadhyay et al. (2020) and aimed to assess the latent construct of sexual and reproductive empowerment among a national sample of American males and females adolescents and young adults (AYAs) aged 15–24 years. This scale contains the following domains: comfort talking with a partner (three questions); choice of partners, marriage, and children (three questions); parental support (4 questions); sexual safety (4 questions); self-love (4 questions); the sense of future (2 questions); and sexual pleasure (3 questions). The total score could range from 0 to 92. The items can be self-administered, and on average, AYAs could answer all items in less than 2 min. The baseline results demonstrated that sexual and reproductive empowerment was associated with access to sexual and reproductive health services and information, and also at 3-month follow-up was moderately associated with the use of desired contraceptive methods. In contrast to most reproductive empowerment measures, this scale can also be used among men and boys [11].

#### Reproductive Autonomy Scale

As a multi-dimensional scale, Reproductive Autonomy Scale (RAS) was developed and validated in the USA to measure “reproductive autonomy” among women. This scale is comprised of 14 items and three subscales. Reproductive autonomy was defined as women’s power to decide about and exercise control on issues related to using contraception, pregnancy, and childbearing. The participants were selected from the family planning and abortion facilities in the United States. Three subscales of the scales were freedom from coercion (five questions), communication (five questions), and decision-making (four questions). The study found a reverse association between freedom from coercion and communication subscales with unprotected sex [24].

#### Reproductive decision-making agency

Hinson et al. (2019) developed and validated the reproductive decision-making agency scale among Nepalese women aged 15–49. The 17-item scale attempts to measure women’s decision-making over reproductive behaviors in three domains, including women’s agency in using family planning methods, agency in choosing the method of family planning, and agency in choosing the time of getting pregnant. In this study, women whose husbands or other relatives rather than themselves mainly made decisions on reproductive behaviors were considered the lowest agency. In contrast, women reporting sole or joint decision makingwere categorized as the medium and high agency, respectively. The scale’s scores varied between three and nine, the higher scores representing the higher agency. This scale can be applied to assess a range of reproductive outcomes, particularly those related to reproductive control.

#### Women’s and Girls’ Empowerment in Sexual and Reproductive health (WGE-SRH)

WGE-SRH was developed by Moreau, Karp, et al. (2020) in three African countries, Ethiopia, Uganda, and Nigeria, to provide a cross-cultural scale. This 21-items scale attempts to assess the existence of choice and exercise of choice across the three domains related to sex, using contraception, and pregnancy. Participant’s agreement or disagreement with each item scored from 1 to 10. The results showed that women who indicated higher scores on the contraceptive choice subscale are more likely to use contraception. Moreover, higher scores on the sexual exercise scale were associated with a higher possibility of volitional sex [13].

#### A short-form Reproductive Coercion Scale (RCS)

This 5-item measure was derived from the Reproductive Coercion Scale (RCS) by McCauley et al. (2017). The scale was validated in two longitudinal randomized controlled trials conducted on young English- or Spanish-speaking women aged 16–29 in the USA. These five questions constructed two subscales: pregnancy coercion (three items) and condom manipulation (two items). Items include dichotomous (yes/no) answers. The short form of scale was useful in recognizing women who endorse low levels of reproduction coercion. This scale is particularly sensitive to identifying women who experience less common forms and multiple forms of reproduction coercion. Furthermore, this scale would provide a rapid assessment of reproductive coercion in clinics.

#### Sexual Assertiveness Scale (SAS)

SAS was developed to measure women’s understanding over the three subscales of assertiveness regarding initiation of sex, refusal of sex, and prevention of sexually transmitted disease/pregnancy (STD-P) with a regular partner. It comprises 18 items rated on a 5-point response format with anchors of 0 (Never) and 4 (Always). The higher scores on the scale, the higher sexual assertiveness is predicted. The SAS was developed and validated in a sample of young American women ages 16–29. After 6 and 12 months intervals, test–retest reliabilities were assessed [5].

#### Spanish version of Hurlbert Index of Sexual Assertiveness

Antos-Iglesias and Carlos Sierra (2010) adapted the Hurlbert Index of Sexual Assertiveness (Hurlbert, 1991) among the Spanish community. The psychometric analysis was conducted among 400 Spanish men and 453 women who had a partner for at least six months. The original scale was composed of 25 items, ranging from 1 (Never) to 5 (Always). The total scores were between 0 to 100. The higher scores represent the higher sexual assertiveness. The exploratory and confirmatory factor analyses identified a 19-item structure with two correlated factors (Initiation and No shyness/Refusal). Six items from the original version were eliminated. Finally, the Spanish version showed satisfactory psychometric characteristics [32].

#### Sexual Assertiveness Questionnaire (SAQ)

SAQ was derived from the Sexual Assertiveness Scale (Morokoff and colleagues, 1997) by Loshek and Terrell (2014) to provide a scale that does not include the condom insistence. The underlying hypothesis was although the sexual assertiveness scale encompasses condom insistence, it might not be administered to women at all life stages or in various kinds of relationships. The final scale comprises 18 items and three subscales, including the ability to initiate and communicate across desired sex, the ability to refuse unwanted sex, and the ability to talk about sexual history and risk. Response choices included a 7-point scale ranging from 1 (strongly disagree) to 7 (strongly agree). The results demonstrate satisfactory psychometric properties [26].

#### Sexual Pressure Scale (SPS)

This 19-items scale aimed to measure gender-stereotypical expectations engaging in sexual behaviors. This study hypothesized that sexual pressure is associated with HIV sexual risk behavior. Scale composed of five factors: Condom Fear, Sexual Coercion, Women’s Sex Role, Men Expect Sex, and Show Trust. Higher sexual pressure was identified through a higher score. The SPS can be used to assess to what extent adherence to gender-stereotypical expectations may limit women’s sexual choices and lead to adverse consequences, such as being less assertive in communicating their desire to reduce risk and being more likely to be engaged in sex with men who are at the higher risk of HIV [7].

#### Sexual Pressure Scale for Women-Revised (SPSW-R)

Jones and Gulick (2009) revised the sexual pressure scale (Jones, 2006) to improve its reliability. The study was carried out on a sample of young adult urban women. The reliability and confirmatory factor analysis using structural equation modeling resulted in 18 items with higher reliability than the original scale. After eliminating the Condom Fear factor, a 4-factor model encompassing Show trust, Women’s sex role, Men expect sex, and Sexual coercion was remained [8].

#### Sexual Relationship Power Scale (SRPS)

This measure was designed by Pulerwitz et al. (2000) to address interpersonal power in sexual decision-making. SRPS consists of 23 items and two subscales, Relationship Control (RC) and Decision-Making Dominance (DM). RC subscale encompasses fifteen,and DM is composed of eight questions. The totalscore was ranged from 8 to 24. Lower scores on SRPS were associated with higher physical violence and lower consistent use of a condom [6].

#### Sexual Relationship Power Scale (SRPS) among adolescent girls and young women (AGYW)

This scale was derived from the Relationship Control subscale of the SRPS and then validated among AGYW who were at the risk of HIV in Kenya. The original subscale consisted of 15 items. A modified scale was extracted after removing three items related to condom use, resulting in 12 items in total. Participants were asked to express to what extent they agree or disagree with each item on a 4-point Likert scale. The results showed that AGYW with higher relationship power were less likely to experience sexual violence and more likely to use a condom and have knowledge of partner’s HIV status [29].

#### Sexual Relationship Power equity (SRP equity)

SRP equity is a South African adaptation of the Sexual Relationship Power, originally developed by Pulerwitz et al. in 2000 [6]. Over the community-based cohorts, 235 young men and women aged 16–24 years completed this questionnaire. Follow-up study performed six months later. The original SRPS consists of 13 questions. Participants answered on a 4-point Likert scale for each item, ranging from (‘strongly agree’ to ‘strongly disagree’). Higher scores representing greater equity in sexual relationship power. Finally, a 8-item scale for women and a 9-item scale for men were constructed. SRP equity was associated with higher education and no recent partner violence [30].

#### Women Autonomy Measurement Scale

This scale was developed by Bhandari et al. (2014) to provide a validated scale for measuring Nepalese women’s autonomy as one of the predictors of using maternal health care services. The 23 items were answered on a 3-point scale anchored with zero (not necessary), one (useful not essential), and two (essential). Three subscales, including decision-making autonomy, financial autonomy, and freedom of movement, constitute the scale. The Autonomy Measurement Scale showed appropriate psychometric characteristics and introduced a valid and standard scale for assessing women’s autonomy in developing countries [27].

#### Women’s Empowerment on Demographic and Health Surveys: indicators for health dimension

Using Demographic and Health Surveys (DHS) from nineteen countries in four African regions, a scale composed of 26 indicators was developed to assess different dimensions of women empowerment, including economic, socio-cultural, education, and health. Access to healthcare composes distance, money, and permission. For instance, items such as: whether women have the “access” or “financial constraints” to make beneficial health choices were included. If women reported difficulties accessing healthcare services, they were assigned a 0 score; otherwise, women were scored 1. This scale provided region-specific indicators of women empowerment in Sub-Saharan Africa [31].

## Discussion

To the best of our knowledge, this is the first review that systematically appraised and summarized the measurement properties of validated women empowerment scales in sexual and reproductive health and also assessed the methodological quality of the included studies. The study results contributed to present a comprehensive picture of the main developments in women reproductive empowermentmeasuresments. Although women empowerment is a broad concept with various domains at different levels, few validated scales exist to measure them. Some domains such as decision-making, freedom of coercion, and communication with a partner were measured more often, whereas others got less attention. It is possibly because most studies concentrated on the individual agency and agency within intimate partnerships (i.e., immediate relational agency). Various domains assessed in the included studies may represent the complex and multi-faceted nature of women empowerment [23]. On the other hand, distant agency focusing on structural empowerment received less attention. Only one study evaluated women's empowerment at the structural level [31]. In this study, Asaolu et al. (2018) investigated healthcare access as a significant contributor to women’s empowerment. The healthcare domain included three variables of distance, money, and permission [31]. They hypothesized that social norms hindering women from going out without somebody’s companionship, financial constraints, farther distance, poor road conditions, or unreliable/no transportation could influence women’s access to healthcare [31]. Women empowerment is a multi-level concept, and social and structural obstacles hinder many women from exercising agency beyond the barriers they face in their marriages or families. Researchers highlighted the importance of embedding the macro-level factors in the definition and measurement of women empowerment [33]. Many factors at the various intrapersonal, interpersonal, and ecological levels determine the degree that a woman is empowered [33]. Social, economic, and cultural systems that operate at the uppermost level play an essential role in shaping the parameters of empowerment in specific contexts. For instance, availability and accessibility of health services, women’s position in the society, the level of power that women can impose in their relationships with their male partners, and cultural expectations of women, effectively influence women empowerment regardless of their individual or household characteristics. Thus, designing scales to measure these structural factors is crucial to understanding reproductive empowerment [3].

All included studies except one used standardized measures that can be applied in other contexts. Comparing women empowerment across the countries would be possible through standardized scales such as autonomy, decision-making, and communication with a partner [34]. Although standards measures are more likely to compare various populations in different cultures, context-specific scales can provide opportunities to reflect women’s lived experiences in contexts in which they live and also allow us to compare the status of their empowerment with peers [35]. In studies that adapted a scale in the new context, some items were removed or substituted by others, indicating the contextual spirit of women empowerment and this fact that dominant beliefs, practices, and values can influence women empowerment. So, probably factors constituting the women empowerment are not similar in different contexts [36, 37]. However, exploring how women in other countries experience empowerment is possible through adapting the existing scales in other contexts to compare women’s situations across the countries.

Sexual Relationship Power Scale and sexual assertiveness scale were most examined as three studies used each of these scales. All included studies focused on women and girls who were in a sexual relationship. Although this enables using scales that measure household, family-in-law, and financial issues of family and capturing the power balance between girls and women and their sexual partners, none of the studies address the never-married women highlighting a gap in developing suitable scales for assessing girls, singles, widows and never-married women. Some studies included men, providing a comparison between women and men's attitudes over women empowerment.

It should be mentioned that all included studies were based on cross-sectional data, limiting the assessment of temporal ordering. A significant concept of women empowerment is the process, emphasizing the changes from one state to another over time [10]. Women’s levels of power can transform over time [28]. Thus, considering changes in the state of women empowerment over time is vital.

Assessing the scales' quality showed that content validity, construct validity, and internal consistency were the most common properties evaluated. Just three studies assessed test–retest reliability. Consequently, their stability to apply to other contexts is doubtful. Rigorous psychometric assessment of the scales is vital. Because poor validity and reliability can endanger the risk of correct evaluation and diagnosis of scales, consequently leading to misinterpretation and inaccurate research findings [38]. In this review, most of the studies achieved moderate or high quality, indicating the appropriate methodology. It appears that the geographic distribution of validated scales is limited to the USA and some African countries. The lack of administration of these scales across various contexts could lead to inadequate external validity [39].

These findings give insights to develop new scales covering more domains of women reproductive empowerment or validate the currently available measurements in various settings on diverse samples.

### Limitations

This systematic review's main focus was finding quantitative measures of women empowerment in sexual and reproductive health, so studies that characterize scales and domains without reporting the development and psychometric analysis were not included. Another limitation of this study is publication bias as the inclusion criteria just considered peer-reviewed articles and excluded gray literature, non-peer-reviewed reports, books, and dissertations. Additionally, including only articles in English may lead to language bias.

## Conclusion

Some dimensions, namely the structural dimension of women empowerment, are being ignored in the existing scales. Including the diverse populations and samples to develop and refine women empowerment’s measurements would facilitate measuring variations in the contexts in which reproductive empowerment is evolved. This study highlighted the necessity of designing and developing comprehensive measures to address the various dimensions of women reproductive empowerment at different levels and in diverse contexts.

## Data Availability

The datasets generated and/or analyzed during the current study are not publicly available as all data are included in the manuscript body, but are available from the corresponding author on reasonable request.
